# (4-Chloro­phen­yl)(3,8-dibromo-2-hydr­oxy-7-meth­oxy-1-naphth­yl)methanone

**DOI:** 10.1107/S1600536810015527

**Published:** 2010-05-08

**Authors:** Ryosuke Mitsui, Shoji Watanabe, Atsushi Nagasawa, Akiko Okamoto, Noriyuki Yonezawa

**Affiliations:** a Department of Organic and Polymer Materials Chemistry, Tokyo University of Agriculture & Technology, 2-24-16 Naka-machi, Koganei, Tokyo 184-8588, Japan

## Abstract

In the title compound, C_18_H_11_Br_2_ClO_3_, an intra­molecular O—H⋯O=C hydrogen bond occurs, forming a six-membered ring. The naphthalene ring system and the benzene ring make a dihedral angle of 57.36 (9)°. The central carbonyl C—(C=O)—C group is twisted away from the naphthalene ring system and the benzene ring by 18.61 (15) and 26.25 (16)°, respectively. In the crystal structure, two inter­molecular Br⋯Cl close contacts [3.4927 (7) and 3.4325 (7) Å] are observed.

## Related literature

For related structures, see: Mitsui *et al.* (2009 ▶); Mitsui, Nakaema, Nagasawa *et al.* (2010 ▶); Mitsui, Nakaema, Noguchi, Okamoto & Yonezawa (2008 ▶); Mitsui, Nakaema, Noguchi & Yonezawa (2008 ▶); Mitsui, Nagasawa, Watanabe *et al.* (2010 ▶). For information on halogen⋯halogen contacts, see: Moorthy *et al.* (2002 ▶); Pedireddi *et al.* (1994 ▶); Saruma & Desiraju (1986 ▶).
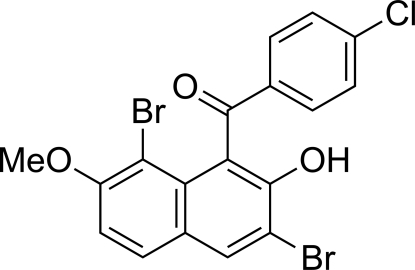


## Experimental

### 

#### Crystal data

C_18_H_11_Br_2_ClO_3_
*M* *_r_* = 470.54
Monoclinic, 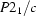

*a* = 12.1513 (2) Å
*b* = 10.06343 (18) Å
*c* = 13.8936 (3) Å
β = 103.675 (1)°
*V* = 1650.79 (5) Å^3^
*Z* = 4
Cu *K*α radiation
μ = 7.85 mm^−1^
*T* = 193 K
0.30 × 0.25 × 0.20 mm

#### Data collection

Rigaku R-AXIS RAPID diffractometer
Absorption correction: numerical (*NUMABS*; Higashi, 1999 ▶) *T*_min_ = 0.189, *T*_max_ = 0.308
29373 measured reflections
3022 independent reflections
2940 reflections with *I* > 2σ(*I*)
*R*_int_ = 0.068

#### Refinement

*R*[*F* ^2^ > 2σ(*F* ^2^)] = 0.028
*wR*(*F* ^2^) = 0.076
*S* = 1.11
3022 reflections
219 parameters
H-atom parameters constrained
Δρ_max_ = 0.60 e Å^−3^
Δρ_min_ = −0.64 e Å^−3^

### 

Data collection: *PROCESS-AUTO* (Rigaku, 1998 ▶); cell refinement: *PROCESS-AUTO*; data reduction: *CrystalStructure* (Rigaku/MSC, 2004 ▶); program(s) used to solve structure: *SIR2004* (Burla *et al.*, 2005 ▶); program(s) used to refine structure: *SHELXL97* (Sheldrick, 2008 ▶); molecular graphics: *ORTEPIII* (Burnett & Johnson, 1996 ▶); software used to prepare material for publication: *SHELXL97*.

## Supplementary Material

Crystal structure: contains datablocks I, global. DOI: 10.1107/S1600536810015527/is2542sup1.cif

Structure factors: contains datablocks I. DOI: 10.1107/S1600536810015527/is2542Isup2.hkl

Additional supplementary materials:  crystallographic information; 3D view; checkCIF report

## Figures and Tables

**Table 1:** Hydrogen-bond geometry (Å, °)

*D*—H⋯*A*	*D*—H	H⋯*A*	*D*⋯*A*	*D*—H⋯*A*
O2—H2*O*⋯O1	0.83	1.85	2.585 (3)	146

**Fig. 1.**
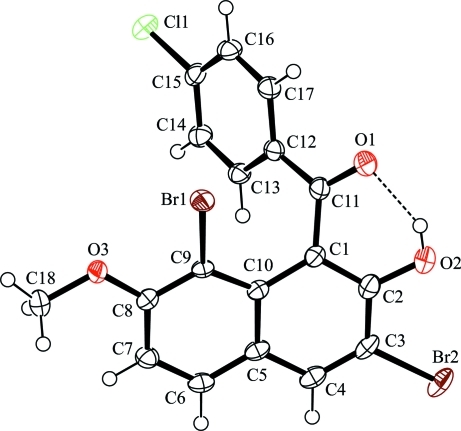
The molecular structure of compound (I), showing 50% probability displacement ellipsoids. The intramolecular hydrogen bond is shown as a dashed line.

**Fig. 2.**
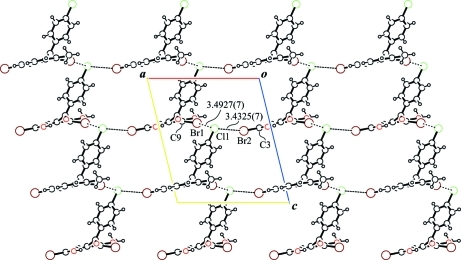
Partial crystal packing diagram of compound (I), viewed down the b axis. Halogen-halogen interactions are shown as dashed lines.

## supplementary crystallographic information

### Comment

Recently, we reported the crystal structures of 1-aroylated 2,7-dimethoxynaphthalenes, 1-(4-chlorobenzoyl)-2,7-dimethoxynaphthalene (Mitsui, Nakaema, Noguchi, Okamoto & Yonezawa, 2008), (4-chlorophenyl)(2-hydroxy-7-methoxynaphthalen-1-yl)methanone (Mitsui, Nakaema, Noguchi & Yonezawa, 2008), (4-chlorophenyl)(2-ethoxy-7-methoxynaphthalen-1-yl)methanone (Mitsui *et al.*, 2009), 1-bromo-8-(4-chlorobenzoyl)-7-hydroxy-2-methoxynaphthalene (Mitsui, Nakaema, Nagasawa *et al.*, 2010) and (8-bromo-2,7-dimethoxy-1-naphthyl)(4-chlorophenyl)methanone (Mitsui, Nagasawa, Watanabe *et al.*, 2010). As a part of our ongoing studies on the synthesis and crystal structure analysis of aroylated naphthalene derivatives, we prepared and analysed the structure of crystal of 2,5-dibromo-4-(4-chlorobenzoyl)-3-hydroxy-6-methoxynaphthalene, (I). The title compound was prepared by electrophilic aromatic bromination reaction of (4-chlorophenyl)(2-hydroxy-7-methoxynaphthalen-1-yl)methanone with bromine.

An *ORTEPIII* (Burnett & Johnson, 1996) plot of (I) is shown in Fig. 1. In the molecule of (I), the intramolecular O—H···O═C hydrogen bond, which forms a six-membered ring including the C═O group and an edge of the naphthalene ring, is present (Table 1). The conformation of these groups resembles to that of (4-chlorophenyl)(2-hydroxy-7-methoxynaphthalen-1-yl)methanone (Mitsui, Nakaema, Noguchi & Yonezawa, 2008). Intriguingly, the central C═O group is twisted away from the naphthalene ring and the benzene ring, and the bromo group at 8-position of naphthalene is out of the least-squares plane of the naphthalene ring. The angles of the C═O bond vector against the least-squares plane of the naphthalene ring and the benzene ring are 18.61 (15) and 26.25 (16)°, respectively. The angle of the C9—Br1 bond vector against the least-squares plane of the naphthalene ring is 14.93 (7)°. This is presumably caused by release of the large steric repulsion brought about by the benzene ring and the bromo group on the naphthalene ring of (I).

In the crystal structure, the contact distances Br1···Cl1 and Br2···Cl1 are 3.4927 (7) and 3.4325 (7) Å, respectively (Fig. 2). These contacts are shorter than the sum of their van der Waals radii (3.60 Å), and the five atoms are arranged nearly linear [C9—Br1···Cl1 = 154.14 (6)°, C3—Br2···Cl1 = 165.35 (7)°], suggesting that there is a possibility for halogen interaction (Saruma & Desiraju, 1986; Pedireddi *et al.*, 1994; Moorthy *et al.*, 2002).

### Experimental

To a solution of (4-chlorophenyl)(2-hydroxy-7-methoxynaphthalen-1-yl)methanone (313 mg, 1.00 mmol) in chloroform (5 ml) was added Br_2_ (483 mg, 3.03 mmol) drop-wise. The reaction mixture was heated at reflux for 2 h, then poured into aqueous 2 M Na_2_S_2_O_3_ (10 ml), and the aqueous layer was extracted with CHCl_3_ (3 × 10 ml). The combined organic layers were washed with 2 M Na_2_S_2_O_3_ (3 × 30 ml) and brine (3 × 30 ml), and dried over MgSO_4_ overnight. The solvent was removed *in vacuo* and the crude material was purified by column chromatography (silica gel, CHCl_3_) to give the title compound (yield 306 mg, 65%). Single crystals suitable for X-ray diffraction analysis were obtained from CHCl_3_ as yellow blocks (m.p. 455.0–455.5 K).

Spectroscopic Data: ^1^H NMR (300 MHz, CDCl_3_) δ 8.10 (s, 1H), 8.04 (s, 1H), 7.75 (d, 1H), 7.58 (d, 2H), 7.31 (d, 2H), 7.17 (d, 1H), 3.95 (s, 3H); ^13^C NMR (75 MHz, CDCl_3_) δ 195.6, 156.3, 152.6, 139.3, 138.3, 134.9, 132.4, 130.3, 129.1, 128.8, 125.8, 118.1, 112.2, 110.5, 105.9, 56.9; IR (KBr): 1670, 1607, 1587, 1495, 1276, 1215, 1096, 782; HRMS (*m/z*): [M + H]^+^ calcd for C_18_H_12_Br_2_ClO_3_, 468.8842 found, 468.8839. Anal. Calcd for C_18_H_11_Br_2_ClO_3_: C 45.95, H 2.36. Found: C 46.23, H 2.39.

### Refinement

All H atoms were located in a difference Fourier map and  were subsequently refined as riding atoms, with O—H = 0.833 Å, C—H = 0.95 Å (aromatic) and 0.98 Å (methyl) Å, and with *U*_iso_(H) = 1.2*U*_eq_(O, C).

### Crystal data

**Table:**

C_18_H_11_Br_2_ClO_3_	*F*(000) = 920
*M**_r_* = 470.54	*D*_x_ = 1.893 Mg m^−^^3^
Monoclinic, *P*2_1_/*c*	Melting point = 455.0–455.5 K
Hall symbol: -P 2ybc	Cu *K*α radiation, λ = 1.54187 Å
*a* = 12.1513 (2) Å	Cell parameters from 28732 reflections
*b* = 10.06343 (18) Å	θ = 3.3–68.2°
*c* = 13.8936 (3) Å	µ = 7.85 mm^−^^1^
β = 103.675 (1)°	*T* = 193 K
*V* = 1650.79 (5) Å^3^	Block, yellow
*Z* = 4	0.30 × 0.25 × 0.20 mm

### Data collection

**Table:**

Rigaku R-AXIS RAPID diffractometer	3022 independent reflections
Radiation source: rotating anode	2940 reflections with *I* > 2σ(*I*)
graphite	*R*_int_ = 0.068
Detector resolution: 10.00 pixels mm^-1^	θ_max_ = 68.3°, θ_min_ = 3.7°
ω scans	*h* = −14→14
Absorption correction: numerical (*NUMABS*; Higashi, 1999)	*k* = −12→12
*T*_min_ = 0.189, *T*_max_ = 0.308	*l* = −16→16
29373 measured reflections	

### Refinement

**Table:**

Refinement on *F*^2^	Secondary atom site location: difference Fourier map
Least-squares matrix: full	Hydrogen site location: inferred from neighbouring sites
*R*[*F*^2^ > 2σ(*F*^2^)] = 0.028	H-atom parameters constrained
*wR*(*F*^2^) = 0.076	*w* = 1/[σ^2^(*F*_o_^2^) + (0.0457*P*)^2^ + 0.9314*P*] where *P* = (*F*_o_^2^ + 2*F*_c_^2^)/3
*S* = 1.11	(Δ/σ)_max_ = 0.001
3022 reflections	Δρ_max_ = 0.60 e Å^−^^3^
219 parameters	Δρ_min_ = −0.64 e Å^−^^3^
0 restraints	Extinction correction: *SHELXL97* (Sheldrick, 2008), Fc^*^=kFc[1+0.001xFc^2^λ^3^/sin(2θ)]^-1/4^
Primary atom site location: structure-invariant direct methods	Extinction coefficient: 0.0162 (4)

### Special details

**Table:**

Geometry. All esds (except the esd in the dihedral angle between two l.s. planes) are estimated using the full covariance matrix. The cell esds are taken into account individually in the estimation of esds in distances, angles and torsion angles; correlations between esds in cell parameters are only used when they are defined by crystal symmetry. An approximate (isotropic) treatment of cell esds is used for estimating esds involving l.s. planes.
Refinement. Refinement of *F*^2^ against ALL reflections. The weighted *R*-factor wR and goodness of fit *S* are based on *F*^2^, conventional *R*-factors *R* are based on *F*, with *F* set to zero for negative *F*^2^. The threshold expression of *F*^2^ > σ(*F*^2^) is used only for calculating *R*-factors(gt) etc. and is not relevant to the choice of reflections for refinement. *R*-factors based on *F*^2^ are statistically about twice as large as those based on *F*, and *R*- factors based on ALL data will be even larger.

### Fractional atomic coordinates and isotropic or equivalent isotropic displacement parameters (Å^2^)

**Table:**

	*x*	*y*	*z*	*U*_iso_*/*U*_eq_	
Br1	0.348845 (18)	0.93221 (2)	0.140206 (18)	0.02724 (13)	
Br2	−0.250748 (19)	0.77531 (3)	0.077383 (18)	0.03487 (13)	
Cl1	0.49341 (5)	0.85290 (6)	0.59621 (4)	0.03239 (17)	
O1	0.18770 (16)	0.61119 (18)	0.17108 (14)	0.0377 (4)	
O2	−0.02937 (15)	0.63660 (18)	0.11024 (13)	0.0357 (4)	
H2O	0.0329	0.5982	0.1198	0.043*	
O3	0.33774 (13)	1.21262 (15)	0.17994 (12)	0.0256 (3)	
C1	0.09899 (18)	0.8215 (2)	0.14949 (15)	0.0222 (4)	
C2	−0.0097 (2)	0.7676 (2)	0.11910 (17)	0.0264 (5)	
C3	−0.10526 (18)	0.8529 (3)	0.10204 (16)	0.0268 (5)	
C4	−0.09270 (18)	0.9868 (3)	0.10944 (16)	0.0268 (5)	
H4	−0.1579	1.0417	0.1004	0.032*	
C5	0.01632 (18)	1.0458 (2)	0.13047 (16)	0.0232 (5)	
C6	0.02945 (19)	1.1856 (2)	0.13389 (17)	0.0266 (5)	
H6	−0.0361	1.2402	0.1217	0.032*	
C7	0.1339 (2)	1.2440 (2)	0.15434 (17)	0.0259 (5)	
H7	0.1408	1.3377	0.1617	0.031*	
C8	0.23139 (18)	1.1645 (2)	0.16441 (15)	0.0216 (4)	
C9	0.21941 (17)	1.0276 (2)	0.15487 (15)	0.0198 (4)	
C10	0.11426 (18)	0.9635 (2)	0.14633 (15)	0.0203 (4)	
C11	0.18698 (19)	0.7270 (2)	0.20076 (17)	0.0249 (5)	
C12	0.26347 (18)	0.7639 (2)	0.29783 (17)	0.0229 (5)	
C13	0.23528 (18)	0.8631 (2)	0.35778 (16)	0.0238 (5)	
H13	0.1678	0.9131	0.3351	0.029*	
C14	0.30470 (19)	0.8895 (2)	0.45010 (17)	0.0261 (5)	
H14	0.2846	0.9559	0.4914	0.031*	
C15	0.40403 (18)	0.8174 (2)	0.48129 (16)	0.0242 (5)	
C16	0.43420 (19)	0.7176 (2)	0.42328 (18)	0.0278 (5)	
H16	0.5024	0.6689	0.4458	0.033*	
C17	0.36288 (19)	0.6908 (2)	0.33217 (18)	0.0268 (5)	
H17	0.3816	0.6217	0.2922	0.032*	
C18	0.3525 (2)	1.3542 (2)	0.18675 (19)	0.0318 (5)	
H18A	0.4326	1.3760	0.1936	0.038*	
H18B	0.3279	1.3873	0.2446	0.038*	
H18C	0.3071	1.3960	0.1267	0.038*	

### Atomic displacement parameters (Å^2^)

**Table:**

	*U*^11^	*U*^22^	*U*^33^	*U*^12^	*U*^13^	*U*^23^
Br1	0.01855 (17)	0.02555 (19)	0.04042 (19)	0.00433 (8)	0.01259 (11)	0.00170 (9)
Br2	0.01849 (17)	0.0547 (2)	0.02984 (18)	−0.01184 (10)	0.00254 (11)	0.00019 (10)
Cl1	0.0229 (3)	0.0404 (4)	0.0307 (3)	−0.0019 (2)	0.0000 (2)	−0.0010 (2)
O1	0.0354 (10)	0.0239 (9)	0.0482 (11)	0.0019 (7)	−0.0014 (8)	−0.0083 (8)
O2	0.0278 (9)	0.0311 (10)	0.0447 (10)	−0.0081 (7)	0.0016 (7)	−0.0024 (8)
O3	0.0196 (8)	0.0220 (8)	0.0346 (9)	−0.0022 (6)	0.0050 (6)	−0.0014 (6)
C1	0.0185 (10)	0.0247 (12)	0.0229 (10)	−0.0011 (9)	0.0038 (8)	−0.0019 (8)
C2	0.0249 (12)	0.0304 (13)	0.0228 (11)	−0.0053 (9)	0.0034 (9)	−0.0009 (9)
C3	0.0150 (10)	0.0423 (14)	0.0219 (10)	−0.0054 (9)	0.0021 (8)	0.0000 (9)
C4	0.0175 (10)	0.0387 (14)	0.0246 (11)	0.0029 (9)	0.0056 (8)	0.0001 (9)
C5	0.0175 (10)	0.0325 (12)	0.0198 (10)	0.0035 (9)	0.0050 (8)	0.0004 (9)
C6	0.0202 (11)	0.0305 (12)	0.0291 (11)	0.0085 (9)	0.0059 (9)	0.0008 (9)
C7	0.0259 (12)	0.0236 (12)	0.0277 (11)	0.0041 (9)	0.0054 (9)	−0.0021 (9)
C8	0.0194 (10)	0.0262 (12)	0.0193 (9)	−0.0001 (8)	0.0046 (8)	−0.0011 (8)
C9	0.0156 (10)	0.0228 (11)	0.0213 (10)	0.0045 (8)	0.0049 (8)	0.0003 (8)
C10	0.0180 (10)	0.0242 (11)	0.0181 (9)	0.0016 (8)	0.0033 (8)	−0.0017 (8)
C11	0.0216 (11)	0.0221 (12)	0.0309 (12)	−0.0018 (9)	0.0063 (9)	−0.0011 (9)
C12	0.0195 (10)	0.0206 (11)	0.0292 (11)	−0.0014 (8)	0.0072 (9)	0.0036 (8)
C13	0.0176 (10)	0.0246 (12)	0.0301 (11)	0.0014 (8)	0.0075 (9)	0.0034 (9)
C14	0.0224 (11)	0.0285 (12)	0.0282 (11)	0.0011 (9)	0.0077 (9)	0.0002 (9)
C15	0.0188 (10)	0.0283 (12)	0.0243 (10)	−0.0046 (9)	0.0031 (8)	0.0024 (9)
C16	0.0191 (11)	0.0291 (13)	0.0352 (12)	0.0035 (9)	0.0063 (9)	0.0051 (9)
C17	0.0235 (11)	0.0243 (12)	0.0333 (12)	0.0019 (9)	0.0079 (9)	−0.0004 (9)
C18	0.0331 (13)	0.0233 (12)	0.0387 (13)	−0.0066 (10)	0.0075 (10)	−0.0012 (10)

### Geometric parameters (Å, °)

**Table:**

Br1—C9	1.894 (2)	C7—C8	1.408 (3)
Br2—C3	1.888 (2)	C7—H7	0.9500
Cl1—C15	1.743 (2)	C8—C9	1.388 (3)
O1—C11	1.237 (3)	C9—C10	1.411 (3)
O2—C2	1.340 (3)	C11—C12	1.492 (3)
O2—H2O	0.8326	C12—C13	1.394 (3)
O3—C8	1.349 (3)	C12—C17	1.398 (3)
O3—C18	1.437 (3)	C13—C14	1.384 (3)
C1—C2	1.398 (3)	C13—H13	0.9500
C1—C10	1.443 (3)	C14—C15	1.387 (3)
C1—C11	1.481 (3)	C14—H14	0.9500
C2—C3	1.418 (3)	C15—C16	1.390 (3)
C3—C4	1.357 (4)	C16—C17	1.381 (3)
C4—C5	1.418 (3)	C16—H16	0.9500
C4—H4	0.9500	C17—H17	0.9500
C5—C6	1.415 (3)	C18—H18A	0.9800
C5—C10	1.424 (3)	C18—H18B	0.9800
C6—C7	1.366 (3)	C18—H18C	0.9800
C6—H6	0.9500		
			
C2—O2—H2O	107.9	C9—C10—C1	124.8 (2)
C8—O3—C18	117.81 (18)	C5—C10—C1	118.17 (19)
C2—C1—C10	119.6 (2)	O1—C11—C1	120.4 (2)
C2—C1—C11	114.8 (2)	O1—C11—C12	118.9 (2)
C10—C1—C11	124.52 (19)	C1—C11—C12	119.97 (19)
O2—C2—C1	123.0 (2)	C13—C12—C17	119.2 (2)
O2—C2—C3	117.3 (2)	C13—C12—C11	122.0 (2)
C1—C2—C3	119.6 (2)	C17—C12—C11	118.7 (2)
C4—C3—C2	121.0 (2)	C14—C13—C12	120.6 (2)
C4—C3—Br2	120.53 (18)	C14—C13—H13	119.7
C2—C3—Br2	118.32 (18)	C12—C13—H13	119.7
C3—C4—C5	121.0 (2)	C13—C14—C15	118.9 (2)
C3—C4—H4	119.5	C13—C14—H14	120.5
C5—C4—H4	119.5	C15—C14—H14	120.5
C6—C5—C4	121.0 (2)	C14—C15—C16	121.7 (2)
C6—C5—C10	119.3 (2)	C14—C15—Cl1	119.13 (18)
C4—C5—C10	119.6 (2)	C16—C15—Cl1	119.13 (18)
C7—C6—C5	121.8 (2)	C17—C16—C15	118.6 (2)
C7—C6—H6	119.1	C17—C16—H16	120.7
C5—C6—H6	119.1	C15—C16—H16	120.7
C6—C7—C8	119.6 (2)	C16—C17—C12	120.9 (2)
C6—C7—H7	120.2	C16—C17—H17	119.5
C8—C7—H7	120.2	C12—C17—H17	119.5
O3—C8—C9	116.51 (19)	O3—C18—H18A	109.5
O3—C8—C7	124.3 (2)	O3—C18—H18B	109.5
C9—C8—C7	119.2 (2)	H18A—C18—H18B	109.5
C8—C9—C10	122.30 (19)	O3—C18—H18C	109.5
C8—C9—Br1	116.24 (16)	H18A—C18—H18C	109.5
C10—C9—Br1	121.15 (17)	H18B—C18—H18C	109.5
C9—C10—C5	117.0 (2)		
			
C10—C1—C2—O2	172.9 (2)	C6—C5—C10—C9	−6.2 (3)
C11—C1—C2—O2	−18.3 (3)	C4—C5—C10—C9	172.45 (18)
C10—C1—C2—C3	−10.9 (3)	C6—C5—C10—C1	175.64 (19)
C11—C1—C2—C3	157.8 (2)	C4—C5—C10—C1	−5.7 (3)
O2—C2—C3—C4	−180.0 (2)	C2—C1—C10—C9	−166.1 (2)
C1—C2—C3—C4	3.7 (3)	C11—C1—C10—C9	26.3 (3)
O2—C2—C3—Br2	4.2 (3)	C2—C1—C10—C5	11.9 (3)
C1—C2—C3—Br2	−172.17 (16)	C11—C1—C10—C5	−155.7 (2)
C2—C3—C4—C5	2.6 (3)	C2—C1—C11—O1	40.3 (3)
Br2—C3—C4—C5	178.36 (16)	C10—C1—C11—O1	−151.6 (2)
C3—C4—C5—C6	177.2 (2)	C2—C1—C11—C12	−129.7 (2)
C3—C4—C5—C10	−1.5 (3)	C10—C1—C11—C12	38.5 (3)
C4—C5—C6—C7	179.8 (2)	O1—C11—C12—C13	−149.6 (2)
C10—C5—C6—C7	−1.6 (3)	C1—C11—C12—C13	20.5 (3)
C5—C6—C7—C8	5.3 (3)	O1—C11—C12—C17	26.3 (3)
C18—O3—C8—C9	177.92 (19)	C1—C11—C12—C17	−163.6 (2)
C18—O3—C8—C7	0.1 (3)	C17—C12—C13—C14	0.1 (3)
C6—C7—C8—O3	176.8 (2)	C11—C12—C13—C14	176.0 (2)
C6—C7—C8—C9	−1.0 (3)	C12—C13—C14—C15	1.3 (3)
O3—C8—C9—C10	174.84 (18)	C13—C14—C15—C16	−1.5 (3)
C7—C8—C9—C10	−7.2 (3)	C13—C14—C15—Cl1	178.49 (17)
O3—C8—C9—Br1	−11.4 (2)	C14—C15—C16—C17	0.2 (3)
C7—C8—C9—Br1	166.54 (16)	Cl1—C15—C16—C17	−179.73 (18)
C8—C9—C10—C5	10.7 (3)	C15—C16—C17—C12	1.2 (3)
Br1—C9—C10—C5	−162.76 (15)	C13—C12—C17—C16	−1.4 (3)
C8—C9—C10—C1	−171.3 (2)	C11—C12—C17—C16	−177.4 (2)
Br1—C9—C10—C1	15.2 (3)		

### Hydrogen-bond geometry (Å, °)

**Table:**

*D*—H···*A*	*D*—H	H···*A*	*D*···*A*	*D*—H···*A*
O2—H2O···O1	0.83	1.85	2.585 (3)	146

## References

[bb1] Burla, M. C., Caliandro, R., Camalli, M., Carrozzini, B., Cascarano, G. L., De Caro, L., Giacovazzo, C., Polidori, G. & Spagna, R. (2005). *J. Appl. Cryst.* **38**, 381–388.

[bb2] Burnett, M. N. & Johnson, C. K. (1996). *ORTEPIII* Report ORNL-6895. Oak Ridge National Laboratory. Tennessee, USA.

[bb3] Higashi, T. (1999). *NUMABS* Rigaku Corporation, Tokyo, Japan.

[bb4] Mitsui, R., Nagasawa, A., Watanabe, S., Okamoto, A. & Yonezawa, N. (2010). *Acta Cryst.* E**66**, o873. 10.1107/S1600536810009463 PMC2983951 21580693

[bb5] Mitsui, R., Nakaema, K., Nagasawa, A., Noguchi, K. & Yonezawa, N. (2010). *Acta Cryst.* E**66**, o676. 10.1107/S1600536810006185 PMC2983574 21580422

[bb6] Mitsui, R., Nakaema, K., Noguchi, K., Okamoto, A. & Yonezawa, N. (2008). *Acta Cryst.* E**64**, o1278. 10.1107/S1600536808017297 PMC2961834 21202910

[bb7] Mitsui, R., Nakaema, K., Noguchi, K. & Yonezawa, N. (2008). *Acta Cryst.* E**64**, o2497. 10.1107/S1600536808039603 PMC2959982 21581458

[bb8] Mitsui, R., Noguchi, K. & Yonezawa, N. (2009). *Acta Cryst.* E**65**, o543. 10.1107/S1600536809004796 PMC2968497 21582202

[bb9] Moorthy, J. N., Natarajan, R., Mal, P. & Venugopalan, P. (2002). *J. Am. Chem. Soc.* **124**, 6530–6531. 10.1021/ja017637i 12047162

[bb10] Pedireddi, V. R., Shekhar Reddy, D., Satish Goud, B., Craig, D. C., Rae, A. D. & Desiraju, G. R. (1994). *J. Chem. Soc. Perkin Trans. 2*, pp. 2353–2359.

[bb11] Rigaku (1998). *PROCESS-AUTO* Rigaku Corporation, Tokyo, Japan.

[bb12] Rigaku/MSC (2004). *CrystalStructure* Rigaku/MSC, The Woodlands, Texas, USA.

[bb13] Saruma, J. A. R. & Desiraju, G. R. (1986). *Acc. Chem. Res.* **19**, 222–228.

[bb14] Sheldrick, G. M. (2008). *Acta Cryst.* A**64**, 112–122. 10.1107/S0108767307043930 18156677

